# The Mucosal Adjuvant Cholera Toxin B Instructs Non-Mucosal Dendritic Cells to Promote IgA Production Via Retinoic Acid and TGF-β

**DOI:** 10.1371/journal.pone.0059822

**Published:** 2013-03-20

**Authors:** Anouk K. Gloudemans, Maud Plantinga, Martin Guilliams, Monique A. Willart, Arifa Ozir-Fazalalikhan, Alwin van der Ham, Louis Boon, Nicola L. Harris, Hamida Hammad, Henk C. Hoogsteden, Maria Yazdanbakhsh, Rudi W. Hendriks, Bart N. Lambrecht, Hermelijn H. Smits

**Affiliations:** 1 Department of Pulmonary Medicine, Erasmus Medical Center, Rotterdam, The Netherlands; 2 Laboratory of Immunoregulation and Mucosal Immunology, Department of Respiratory Medicine, University of Ghent, Ghent, Belgium; 3 Department of Parasitology, Leiden University Medical Center, Leiden, The Netherlands; 4 Bioceros B.V., Utrecht, The Netherlands; 5 Laboratory of Intestinal Immunology, Ecole Polytechnique Fédérale de Lausanne, Lausanne, Switzerland; 6 Department of Molecular Biomedical Research, VIB, Ghent, Belgium; Institut national de la santé et de la recherche médicale (INSERM), France

## Abstract

It is currently unknown how mucosal adjuvants cause induction of secretory immunoglobulin A (IgA), and how T cell-dependent (TD) or -independent (TI) pathways might be involved. Mucosal dendritic cells (DCs) are the primary antigen presenting cells driving TI IgA synthesis, by producing a proliferation-inducing ligand (APRIL), B cell activating factor (BAFF), Retinoic Acid (RA), TGF-β or nitric oxide (NO). We hypothesized that the mucosal adjuvant Cholera Toxin subunit B (CTB) could imprint non-mucosal DCs to induce IgA synthesis, and studied the mechanism of its induction. *In vitro*, CTB-treated bone marrow derived DCs primed for IgA production by B cells without the help of T cells, yet required co-signaling by different Toll-like receptor (TLR) ligands acting via the MyD88 pathway. CTB-DC induced IgA production was blocked *in vitro* or *in vivo* when RA receptor antagonist, TGF-β signaling inhibitor or neutralizing anti-TGF-β was added, demonstrating the involvement of RA and TGF-β in promoting IgA responses. There was no major involvement for BAFF, APRIL or NO. This study highlights that synergism between CTB and MyD88-dependent TLR signals selectively imprints a TI IgA-inducing capacity in non-mucosal DCs, explaining how CTB acts as an IgA promoting adjuvant.

## Introduction

Secretory immunoglobulin A (SIgA) is abundantly present at mucosal surfaces of the gastrointestinal and respiratory tract. Here, SIgA, prevents pathogens and commensal bacteria from binding to epithelial cells, it prevents ingested or inhaled allergens to cause immunopathology and it neutralizes toxins, thus broadly acting to maintain homeostasis in the gut and lung [1]–[4]. Inducing IgA synthesis might be beneficial in a number of immune-mediated mucosal diseases like asthma. Lack of IgA is associated with increased rates of sensitization to inhaled and ingested allergens [5], [6], whereas adoptive transfer of allergen-specific IgA or IgA producing B cells in mice can protect from allergic disease [7], [8]. If we are to exploit the full potential of IgA as an immunomodulatory immunoglobulin in mucosal diseases such as asthma, we need to understand better how IgA synthesis is regulated and how we can promote the synthesis of IgA through the use of adjuvants.

IgA synthesis is regulated by both T cell-dependent (TD) and T cell-independent (TI) pathways. In TD IgA synthesis, antigen specific naïve B cells differentiate into IgA^+^-committed B cells upon stimulation by CD40L expressed on activated T cells and TGF-β expressed by multiple cell types. Alternatively, TI IgA synthesis is induced in polyclonal naïve B cells by dendritic cell (DC)- and epithelial cell- derived molecules, such as proliferation-inducing ligand (APRIL), B cell activating factor (BAFF), Retinoic Acid (RA), TGF-β or nitric oxide (NO) [9]–[11]. Mucosal DCs, found in Peyer’s Patches (PP) and lamina propria of the gut or in the lung epithelium and lamina propria [12], are the primary antigen presenting cells that can drive TI (canonical) IgA class switching. Importantly, mucosal conditioning of DCs occurs via tissue-derived factors, such as RA and TGF-β, but also by (commensal) bacteria expressing Toll-like receptor (TLR) ligands [13]–[15].

We hypothesized that there might exist mucosal adjuvants that imprint non-mucosal DCs to stimulate humoral IgA responses through instructive signals that closely mimick those found during residence of mucosal DCs in their natural mucosal environment. We focused on the TLR-independent molecule Cholera Toxin subunit B (CTB), produced by the bacterium *Vibrio cholerae.* Cholera toxin (CT) contains a toxic ADP-ribosyltransferase subunit A, linked to a pentamer of non-toxic B carrier subunits. CTB was shown to bind specifically to GM1-ganglioside (GM1), a receptor expressed on the membrane of most types of epithelial cells, but also on various hematopoietic cells. CTB is widely used as a mucosal adjuvant, stimulating tolerance to co-administered antigen [16], [17]. In a mouse model, CTB enhanced IgA responses against inhaled allergens [8]. Here we studied whether CTB can prime non-mucosal DCs to induce IgA production, and whether similar molecular signals are involved in the cellular communication between DCs and B cells as described for TI IgA synthesis induced by mucosal PP DCs.

## Results

### CTB+LPS-primed Bone Marrow Derived DCs Promote IgA Production in vitro

**Figure 1:**
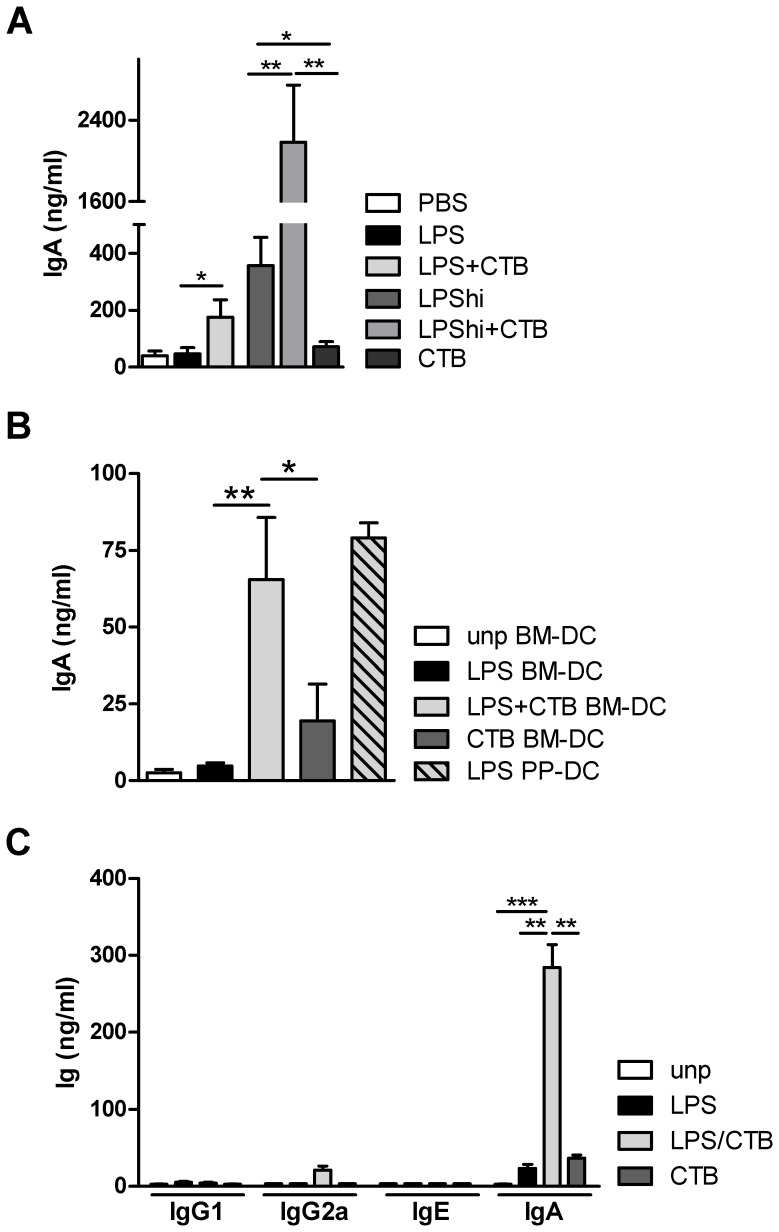
CTB/LPS-primed BM-DCs promote IgA production in vitro. BM-derived DCs were cultured for 8 days with GMCSF, pulsed overnight with PBS, LPS (1 ng/ml) +/− CTB (10 µg/ml) or CTB alone, and then co-cultured with splenic B cells (ratio 1∶1) and anti-IgM Fab-fragments (10 µg/ml). After 7 days, IgA levels were determined by ELISA. (A) BM-DC and B cells of Balb/c background were used for the co-culture. In addition to the conditions described, a high concentration (100 ng/ml) of LPS was used to pulse the BM-DC (B) Besides BM-DC, PP-DC were used to put in co-coculture with B cells (all of C57/Bl6 background) (C) In addition to IgA, also other Immunoglobulin isotypes were measured in supernatant of cocultures (Balb/c) by ELISA. Mean+sem of at least 5 individual experiments are shown. * P<0.05, ** P<0.01, *** P<0.001.

To study whether CTB could prime non-mucosal DCs to induce IgA production, we employed an *in vitro* co-culture system in which bone marrow derived (BM)-DCs were cultured with splenic B cells (Balb/C background), in a one-to-one ratio for seven days (adapted from [18]). DCs were grown in GM-CSF, mainly generating inflammatory-type DCs. To address the impact of TLR signaling and mucosal adjuvants on DC function, the DCs were first exposed to LPS with or without CTB. Significant levels of polyclonal IgA (∼200 ng/ml) were measured in supernatant when B cells were co-cultured with LPS (1 ng/ml)+CTB-pulsed DCs, compared to low levels of IgA (<80 ng/ml) in the control conditions CTB- or LPS-primed DCs. Interestingly, although BM-DCs primed with 100 times more LPS (100 ng/ml; LPS^hi^) were able to induce a significant IgA production, the addition of CTB during priming dramatically further enhanced the IgA production by co-cultured B cells (**Figure 1a**). Surface IgA staining confirmed the generation of IgA positive splenic B cells (∼3-fold increase for LPS+CTB condition) (data not shown). Similar data were obtained when using naive B cells (CD43^−^ splenocytes) (Figure S1a) or BM-derived conventional DCs generated in the presence of Flt3L (Figure S1b), suggesting these findings were not due to expansion of a pre-existing memory IgA-class switched B cell population or was unique to inflammatory DCs. IgA induction by non-mucosal BM-DCs was compared to bona fide mucosal PP-DCs, representing the natural IgA inducing capacity of cells specialized in promoting this response. To obtain sufficient amounts of PP-DCs, C57Bl/6 mice were first injected subcutaneously with a B16 Flt3-L secreting melanoma cell line [19] to increase recovery of ex vivo DCs. The priming of BM-DC by LPS+CTB resulted in non-mucosal-DCs that were as potent as that of *ex vivo* purified mucosal DCs obtained from the PP (**Figure 1b**). The *ex vivo* PP-DCs preparation contained ∼4% CD19^+^ B cells, which may harbor some memory IgA producing B cells, potentially influencing the IgA levels found in these cultures. However, there was evidence of active class switching in naïve B cells. In cultures with total B cells (CD19+) or with naïve B cells (CD43^−^), the enzyme activation-induced deaminase (AID), involved in antibody class switching, was upregulated in the condition of LPS+CTB pulsed DCs (Figure S1c). As in general low levels of other immunoglobulins (IgG1, IgG2a, IgE) were detected in all conditions (**Figure 1c**), this shows that CTB has a predominant IgA promoting effect. Addition of an adenylate cyclase inhibitor did not affect the induction of IgA by LPS+CTB-pulsed DCs, excluding the contribution of Cholera Toxin subunit A traces in this process (data not shown). Importantly, B cells exposed to CTB or LPS+CTB (either followed by culture with mature DCs or not) did not produce IgA, indicating that CTB did not act on B cells directly (data not shown).

These results were reproducible using cells obtained from mice of both C57Bl/6 and Balb/C genetic background, although IgA levels were generally (>3 fold) lower in C57Bl/6 cells (**Figure 1a** **vs 1b**). Throughout the paper, cultures were done with cells isolated from Balb/C mice, except for cultures with KO cells as these mice are of C57Bl/6 background.

### MyD88 Driven Pathways Synergize with CTB Priming to Drive IgA Promoting DCs

**Figure 2:**
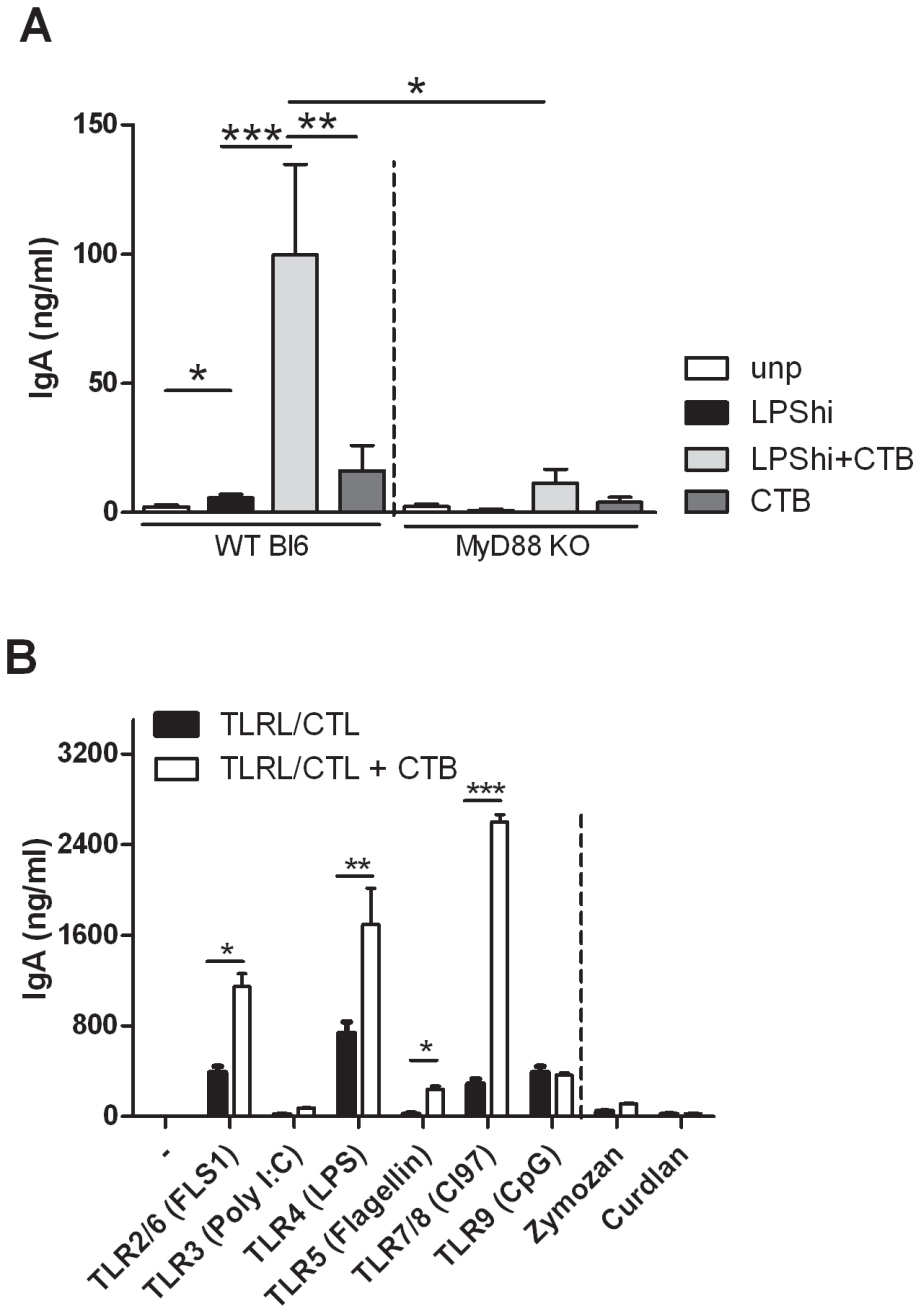
MyD88 driven pathways synergize with CTB priming to drive IgA promoting. *DCs.* (A) BM-DCs from WT or MyD88^−/−^ mice (C57/Bl6 background) were pulsed with PBS, LPShi (100 ng/ml), LPShi+ CTB (10 µg/ml) or CTB alone and co-cultured as described at Figure 1. (B) BM-DCs were pulsed with PBS, or different TLR ligands (Poly I:C 25 µg/ml, LPS 100 ng/ml, Flagellin 1 µg/ml, FLS1 10 µg/ml, Cl97 1 µg/ml, CpG 2.5 µg/ml) or CTLs (Zymozan 10 µg/ml, Curdlan 150 µg/ml) with and without CTB, followed by co-cultures with splenic B cells. Data of one representative experiment out of three is shown. * P<0.05, ** P<0.01, *** P<0.001.

The fact that CTB-primed DCs did not induce IgA production by co-cultured B cells whereas CTB+LPS-primed DCs readily did, suggests a synergistic effect of CTB- and LPS activated pathways in instructing DCs to adopt a mucosal phenotype. LPS activates the TLR4 receptor, which signals through the downstream adaptor molecule MyD88. The IgA promoting effect of LPS+CTB primed DCs was abrogated when wild type B cells and MyD88-deficient DCs (C57Bl/6 background) were cultured together, even when the high concentration LPS (100 ng/ml) was used, pointing at a crucial involvement of MyD88 (**Figure 2a**).

Next we questioned whether co-activation of the DCs via other Pattern Recognition Receptor (PRR) pathways was also able to promote CTB-driven IgA induction by DCs. Therefore DCs were stimulated with different TLR ligands (FLS1 (TLR2/6), poly I:C (TLR3), LPS (TLR4), flagellin (TLR5), CL97 (TLR7/8), or CpG (TLR9)), or non-TLR PRRs such as C-type lectin ligands (CTLs) Zymosan (TLR2/Dectin) and Curdlan (Dectin-1) with or without CTB, followed by co-cultures with splenic B cells (Balb/C). Interestingly, high level microbial priming of BM-DCs by several TLR ligands alone, e.g. LPS (high dose, 100 ng/ml)), FLS1, CL97 and CpG, was sufficient to induce IgA production (**Figure 2b**, black bars). Importantly, CTB strongly further enhanced the IgA induction by DCs primed with MyD88-dependent TLRs; but only slightly or not at all for DCs primed with the MyD88-independent TLR ligand (poly I:C) or the non-TLR-dependent CTLs (Zymozan and Curdlan) (**Figure 2b** **)**. Intriguingly, the IgA inducing capacity of DCs primed by CpG was not further enhanced by CTB, although TLR9 activity is also MyD88-dependent.

### Critical Involvement of DC-derived RA and TGF-β

**Figure 3:**
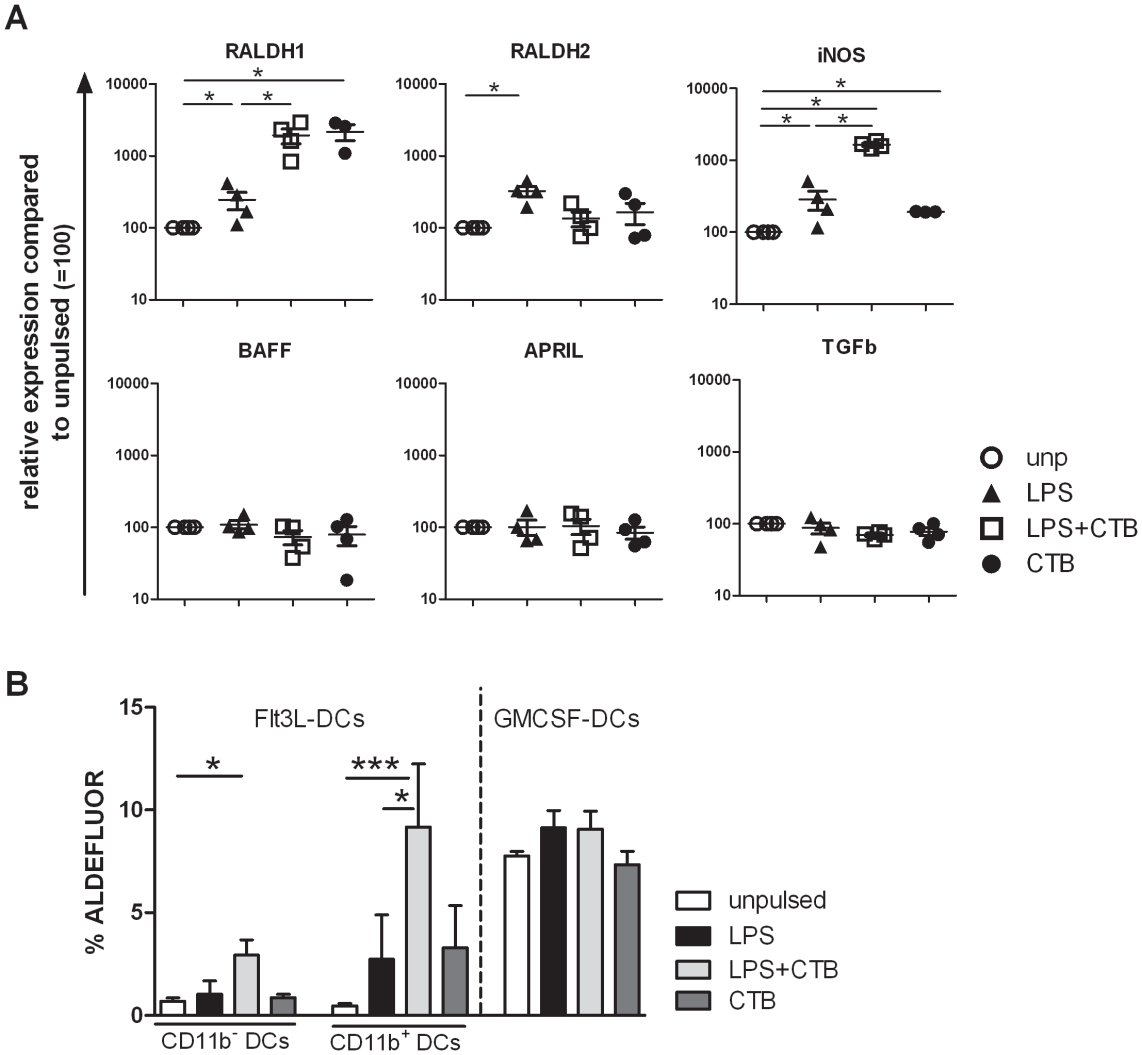
Expression of candidate IgA inducing molecules of BM-DCs. (A) BM-DCs were cultured as described previously and pulsed with PBS, LPS (1 ng/ml) +/− CTB (10 µg/ml) or CTB alone. After 24 hrs the following gene expression was analyzed (A) RALDH1, RALDH2, iNOS, BAFF, APRIL and TGF-β. Expression is normalized against the house keeping gene GAPDH and displayed as relative to conventional unpulsed BM-DCs. Mean+sem of 4 indvidual BM-DC cultures are shown. (B) BM-DCs were generated by culture in GM-CSF or Flt3-L, and pulsed as described. After 24 hours, cells were washed and stained for ALDEFLUOR activity (according to the instructions of the manufacturer), CD11c, CD11b and MHCII expression and analyzed by flowcytometry. The results are a mean+sem from 4 independent experiments. * P<0.05, ** P<0.01, *** P<0.001.

Priming of non-mucosal BM-DCs with CTB in synergy with MyD88-dependent TLR ligands leads to IgA-inducing DCs. Several factors, including NO, RA, TGF-β, APRIL and BAFF can promote IgA production at the intestinal mucosa [9]. To elucidate whether the IgA inducing capacity of CTB-primed BM-DCs was associated with one of these factors, we analyzed gene expression profiles of RA synthesizing enzymes retinal dehydrogenase 1 (RALDH1) and 2 (RALDH2), inducible NO Synthetase (iNOS), TGF-β, BAFF and APRIL in 24 hrs conditioned DCs by PCR from 4 independent Balb/C DC cultures. RALDH1, RALDH2 and iNOS gene expression was slightly but significantly upregulated in LPS-primed DCs 24 hrs post-pulsing, compared to unpulsed DC. Interestingly, LPS+CTB-primed DCs which support IgA responses, express highly elevated levels of RALDH1 and iNOS compared to the other conditions, while BAFF, APRIL and TGF-β gene expression was equally expressed in all conditions (**Figure 3a**). As LPS+CTB-primed DCs and CTB-DCs expressed equal enhanced gene expression levels of RALDH1, we also analyzed ALDEFLUOR activity in both GMCSF-cultured and in FLT3-L-cultured DCs after exposure to CTB or LPS+CTB. In FLT3-L cultured DCs we observed a clear upregulation of ALDEFLUOR activity after priming with LPS+CTB, but not in response CTB only or to the other control conditions (**Figure 3b**). This correlated well with their capacity to induce IgA (Figure S1b) and suggests a differential capacity to enhance ALDEFLUOR activity despite equal enhanced RALDH1 gene expression comparing LPS+CTB and CTB only. Although the activity was most pronounced in CD11b^+^ DCs, the same trend was visible in CD11b^−^ DCs. Surprisingly, GMSCF-cultured DCs did not show any enhanced ALDEFLUOR activity in response to LPS+CTB (**Figure 3b**). But this may be explained by the fact that the basal levels of ALDEFLUOR activity were already extremely high (MFI 100 fold higher compared to Flt3-L DCs) in unprimed GM-CSF DCs, making it difficult to measure any further enhancement. This is probably the result of the continuous exposure to GM-CSF as shown by Yokota et al [20]. Of note, we observed an abolished IgA production by LPS+CTB-primed DCs in medium with UV-irradiated FCS (data not shown), in which serum-derived retinoids were destroyed, confirming an underlying role for DC metabolism of retinal to RA in their capacity to induce IgA in B cells. This mechanism has similarly been described for mesenteric lymph node DCs, at least with respect to the RA-dependent induction of gut homing receptors on T cells [21].

Gene expression of TGF-β was not enhanced, but regulation may occur at the posttranscriptional level. Furthermore, our previous studies point towards an important role for TGF-β in CTB-driven IgA-dependent protective responses against AAI [8]. Subsequent protein expression of TGF-β (measured by, yet inactive, LAP) was slightly but consequently increased in CTB-primed DCs, compared to control DCs (Figure S2).

**Figure 4:**
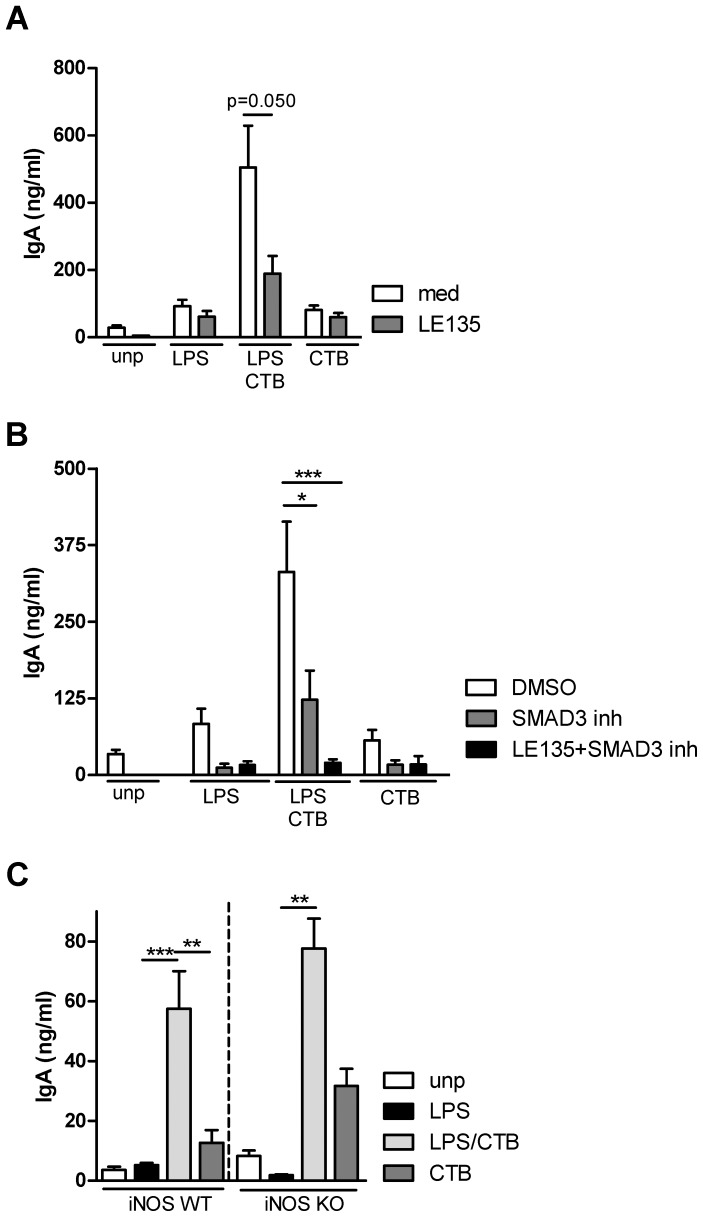
In vitro, RA and TGF-β are responsible for IgA induction by CTB-primed DCs. BM-DCs were pulsed with PBS, LPS (100 ng/ml), LPS+ CTB (10 µg/ml) or CTB alone and co-cultured as described at Figure 1. (A) During the co-culture the RA receptor was blocked by LE135 (1 µM). After 7 days, IgA levels were measured in supernatant. (B) Similar as in A, but here in the presence of SB-431542 (5 µM) (or vehicle) alone to inhibit TGFβ-specific signaling or together with LE135. After 7 days, IgA levels were measured in supernatant. (C) BM-DC and B cells were co-cultured as described, however the DC were WT or deficient for iNOS (C57/Bl6 background). Data shown are mean +/− s.e.m from at least 3 pooled experiments * P<0.05, ** P<0.01, *** P<0.001.

To study the functional involvement of these molecules, *in vitro* experiments were performed in which either the cells were genetically deficient for certain critical mediators or chemical inhibitors were added. When the RA receptor was blocked by the antagonist LE135, we consistently observed a partial inhibition of IgA production by B cells cultured with LPS+CTB-DC (**Figure 4a**). RA receptor inhibitor LE540 or Citral, which block RA production through blockade of the enzymatic activity, resulted in similar inhibition (data not shown). Blocking TGF-β signaling, using SB-431542, significantly decreased (2–4 fold) IgA production by LPS+CTB-primed DCs (**Figure 4b**). Importantly, when both TGF-β signaling and signaling via RA were blocked, the IgA response was completely abrogated (**Figure 4b**). Equal proliferation of CFSE-labeled B cells and similar IgM levels were found for all conditions (data not shown) excluding cytotoxic effects of the inhibitors.

Although mRNA expression for iNOS was strongly elevated in LPS+CTB-primed DCs, blocking NO synthesis by the inhibitor L-NIL only had a marginal effect on IgA production (data not shown). Furthermore, co-cultures of iNOS^−/−^ BM-DCs and WT B cells resulted in similar IgA levels as in WT cultures (**Figure 4c**), indicating that iNOS is not involved in CTB-induced IgA production in our model. Blocking either BAFF and/or BAFFR3 did not affect CTB-induced IgA production (Figure S3a). APRIL gene expression was not induced in LPS+CTB-primed DCs, however APRIL may also be regulated during translation [22]. Therefore, we investigated a possible role for APRIL in mice lacking both receptors for APRIL, the Transmembrane activator and CAML-interactor (TACI) and B cell maturation antigen (BCMA) double deficient mice. Of note, TACI and BCMA are also receptors for BAFF, but BAFF can still signal through a third receptor, the BAFF3 receptor. Surprisingly, co-cultures of TACI/BCMA^−/−^ B cells and LPS/CTB-primed DCs resulted in strongly elevated levels of IgA (>1000 ng/ml) compared to WT control cultures (Figure S3b). These results may suggest that signaling via the receptors TACI and BCMA has a negative influence rather than a promoting function. Alternatively, these results may be explained by highly proliferating cells in which the process of class switching was prevented.

In summary, these data support a dominant role for both TGF-β and RA in driving CTB-induced IgA responses *in vitro*.

### Ex vivo CTB-pulsed DCs Promote IgA Responses in the Lungs via RA and TGF-β

**Figure 5:**
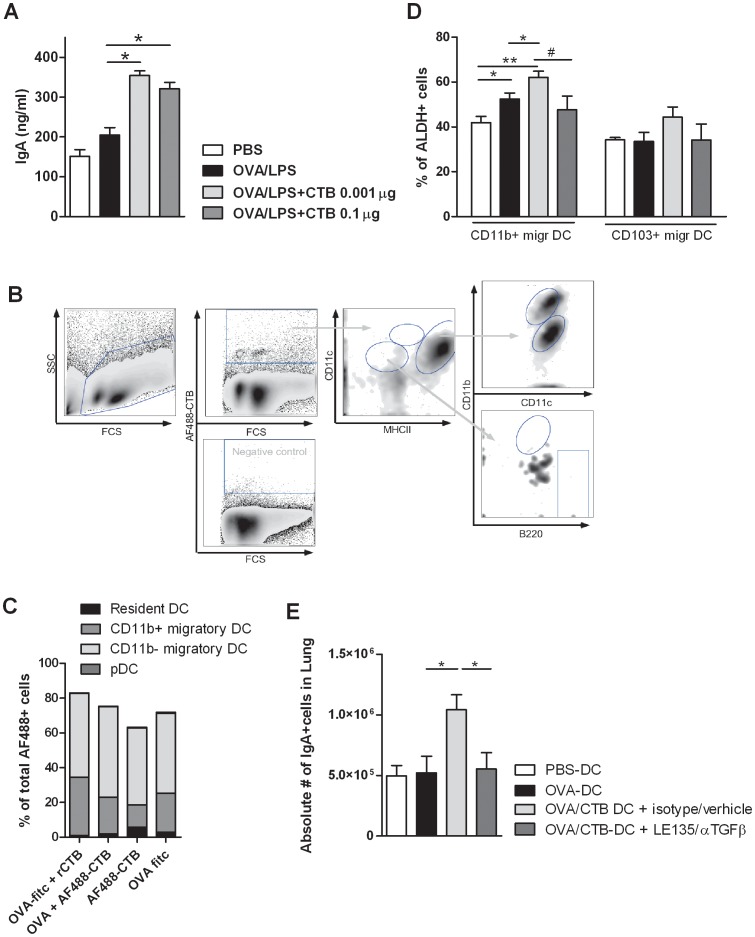
Ex vivo CTB pulsed DCs promote IgA responses in the lungs via RA and TGF-β. PBS, OVA (800 µg/80 µl/mouse), OVA+CTB (0.1 or 0.001 µg/80 µl) or CTB alone were instilled in lungs of naïve mice. (A) After OVA challenge, lung B cells were isolated, cultured and restimulated by LPS (10 µg/ml) for 7 days and IgA levels were determined (B) AF488-labeled CTB (and as a control Fitc-labeled OVA) was used. After 36 hrs, MedLN and lung tissue were studied for the presence of AF488-positive cell populations: Dead cells are excluded based on FSc and SSc; AF488 positive cells selected; migratory cDCs (MHCII^hi^) subdivided into CD11b+ and CD11b- subsets; resident DCs (MHCII^int^); CD11c^lo^MHCII^lo^ cells, subdivided into pDC (B220+) and alveolar macrophages (CD11b+) (C) As in B. Percentage of AF488-positive DC subsets total AF488+ cells in MedLN (D) After 36 hrs, MedLN were isolated. ALDH enzyme activity of different DC subsets was determined using ALDEFLUOR assay, by flowcytometry. (E) BM-DC were generated as described and pulsed with PBS, OVA (100 µg/ml), OVA+CTB (10 µg/ml) or CTB only (10 µg/ml) and the next day intratracheally injected in naïve mice. One day before until 3 days after DC instillation, mice were treated i.p with LE135 and anti-TGFβ antibody (or isotype Ab and DMSO). After one week, the mice were challenged by OVA for 3 consecutive days. One day after challenge, the number of IgA+ cells in digested lungs was determined by flowcytometry. Data are mean +/− s.e.m, of at least 2 individual experiments with 4 mice per group (for D: 12 mice per group, pooled per 3 mice, resulting in 4 datapoints per group). ^#^ p = 0.10 * P<0.05, ** P<0.01, *** P<0.001.

*In vitro*, synergistic signaling of CTB and MyD88 conditioned non-mucosal DCs to promote IgA responses, via TGF-β and RA. We wondered whether it was possible to prime DC *in vivo,* and stimulate IgA responses. To address this question, we decided to administer CTB in the lungs of anesthetized mice via aspiration thought the glottis. Using the pulmonary route of delivery allows consistent delivery of CTB without the need of breeching any physiological barriers through injections. Needle injection through the skin causes damage and inevitable release of damage associated molecular patterns that could influence IgA induction. A single administration of CTB along with the OVA to the airways resulted in increased IgA production by lung B cells that where isolated one day after the last OVA challenge (**Figure 5a**). Previously, in a paper focusing on OVA-induced experimental asthma, we published that adoptive transfer of the *in vitro* generated OVA/CTB-pulsed BM-DC results in increased IgA responses *in vivo* [8]. The OVA (Worthington) used as a model antigen here, contains a sufficient amount of LPS to provide for the necessary MyD88 signaling (Figure S4). Furthermore, we studied whether DCs are prominently targeted by CTB when administered in the airways, as many hematopoietic cells express GM1 [16]. AF488-labeled CTB with or without OVA was administered into the airways of naïve mice, after 36 hrs the AF488^hi^ cells present in the lung-draining lymph nodes were analyzed, using markers that specifically identify the different DC subsets and other APCs (**Figure 5b**) The vast majority of the AF488^hi^ cells (almost 80%) consisted of DCs; both CD11b^+^ and CD11b^−^ migratory DCs (MHCII^hi^CD11c^hi^), resident DCs (MHCII^int^CD11c^hi^), and some pDCs (MHC^lo^CD11c^int^B220^+^CD11b^−^) (**Figure 5c**). Some B cells (MHCII^hi^B220^+^, ∼10%) and only a few T cells (CD3^+^) and macrophages (<1%, MHCII^lo^CD11c^int^CD11b^+^B220^−^) had taken up the labeled CTB (data not shown). The remaining non-migrated AF-488^hi^ cells left behind in the lungs also mainly consisted of DCs, containing equal percentages of resident CD11b^+^ and CD11b^−^ DCs (MHCII^+^CD11c^+^) (data not shown).

As these experiments revealed that CTB also targeted predominantly the DC population in vivo and similarly led to increased IgA induction in the lung, we next aimed to investigate whether CTB modified DC function *in vivo* as it did *in vitro*. Therefore, CTB +/− OVA was instilled in the airways of mice and the RA producing capacity of different DC subsets was studied by flow cytometry at locations of potential interaction between DCs and B cells, i.e. the draining Mediastinal LN (MedLN) and the lung. Aldehyde dehydrogenase (ALDH, catalyzes oxidation of retinal into RA) activity of DCs was determined using ALDEFluor staining kit. Both CD103^+^ and CD11b^+^ migratory DC subsets found in the MedLN showed ALDH enzyme activity, ∼35% and ∼45% of the DCs subsets respectively. But upon OVA+CTB instillation, a significantly higher percentage of migratory CD11b+ DCs (>60%) were positive in the ALDEFluor assay compared to CD11b+ DCs (<50%) from control mice not treated with OVA+CTB (**Figure 5d** **,** for representative dotplots of ALDEFluor expression see Figure S5a/b). In contrast, there were considerably less DCs left behind in the lung that expressed ALDH: 10–20% positive cells for both CD103^+^ and CD11b^+^ DCs, but no differences were observed between the groups (data not shown).

The findings that CTB targets mainly DCs and induces increased IgA via upregulating the expression of the RA synthesizing enzyme on CD11b^+^ DCs *in vivo,* directed us to further study the role of RA and TGFβ in the *in vivo* induction of IgA in CD11b^+^ DCs. The numbers of CD11b^+^ DCs in lung draining LNs are very low, and insufficient numbers of DCs could be sorted from the LNs to perform adoptive transfer experiments. Therefore, BM-DCs which are mainly of the CD11b^+^ type were used for *in vivo* adoptive transfer experiments. As published previously, DCs exposed ex-vivo to OVA+CTB and injected into the airways of naïve mice resulted in increased IgA levels in BAL fluid, increased IgA production by *ex vivo* restimulated lung B cells, and increased number of IgA^+^ cells in lung tissue, compared to mice injected with unpulsed, OVA-pulsed or CTB-pulsed DCs [8]. Using this system, the involvement of both TGF-β and RA in CTB-driven IgA production was revealed, by blocking the function of these factors *in vivo*. Anti-TGF-β antibody and RA receptor blocking agent LE135 were administered one day before until 3 days after the intratracheal administration of OVA +/− CTB-pulsed DCs. After OVA aerosol challenge to boost immunoglobulin production, both the percentage and the absolute number of IgA^+^ cells in the lung were reduced in lung tissue of mice treated with LE135 and anti-TGF-β compared to control mice only receiving solvent and OVA+CTB pulsed DCs (**Figure 5e** **)**.

## Discussion

DC-driven IgA induction has been demonstrated to be a unique feature of mucosal DCs such as lamina propria or PP DCs in the gut [23], [24], expressing several IgA inducing factors, including RA, TGF-β, iNOS, APRIL and/or BAFF [13]. Here we demonstrate that CTB, in conjunction with MyD88-dependent TLR ligands, can imprint non-mucosal BM-DCs to induce strong IgA responses in naïve B cells This involves partly similar IgA-inducing factors as described for mucosal DC, as we identified a dominant positive role for RALDH1 and TGF-β, but not for iNOS, BAFF and APRIL. It has been shown, that also the mucosal factor RA can enhance the IgA inducing capacity of non-mucosal BM-DC, by increasing RALDH2 expression and TGF-β production and being amplified by MyD88 signaling, similar to what we here observe for CTB [19], [25], [26]. An interesting possibility might be that the working mechanism of CTB can be explained via the induction of enhanced RA expression by DCs which may affect DC function in an autocrine loop.

Despite the widespread availability of retinol, RALDH expression is limited to certain cell types. DCs that are located in gut-associated lymphoid tissue (GALT) and express RALDH2 are well known for their RA production and IgA inducing ability. But other DC subsets with RA producing capacity have been described and expression of one of the aldehyde dehydrogenases, RALDH1, 2 or 3, is essential for a cell to be able to catalyze the oxidation of retinal into RA [15], [27], [28]. In lung DC, expression of RALDH1 is more prominent like in our CTB-treated BM-DCs [29]. The fact that we do not find differences in RALDH2 expression of cultured CTB exposed BM-DC, could be due to the GMCSF which is used to generate the BM-DC in vitro. Yokota et al showed that GMCSF is sufficient to markedly induce RALDH2 expression in cultured BMDC, and this could overrule the effect of CTB in our system [20]. Nevertheless, although removal of GM-CSF for 24 hours during pulsing did result in much lower gene expression profiles of RALDH2, we still did not observe an upregulation for RALDH2, only RALDH1 (data not shown), possibly explained by an irreversible effect of GM-CSF stimulation on RALDH2 transcription. Furthermore, CTB only induced similarly enhanced levels of RALDH1 as LPS+CTB, while TGF-β was mostly increased in the LPS+CTB condition. This suggests that a combined action of RALDH and TGFβ is necessary, like recently described by Feng et al for the IgA-inducing activity of RA on BM-DCs [25] Therefore, CTB either partly mimics the effects of RA by inducing similar mucosa-associated factors or acts via the induction of RA itself in non-mucosal DCs.

CTB binds to GM-1 ganglioside, which is present on many different hematopoietic and structural cell types [16]. Despite this wide expression pattern, we showed that CTB administered into the airways mainly targeted DCs and that CTB-loaded DCs migrated to the draining LNs (figure 5b). This finding is supported by other studies in the skin, showing a specific recruitment of DCs towards areas of CT administration [30], [31]. In the lung of mice, several DC subsets have been described: e.g. two major subsets of conventional DCs have been defined based on their CD103 and CD11b expression [32]. Very recently, it was published that in vitro mouse CD11b^high^ lung DC induce IgA more efficiently, than CD103^+^ lung DC [33]. However, it was not clear by what mechanisms this was induced, as the expression of BAFF, APRIL or RALDH1 was not different between the two lung DC subsets. In the gut, specialized DC subsets inducing TI IgA synthesis are iNOS^+^TNF^+^ DCs (tipDCs) [34], and the recently described CD11b^+^ DCs expressing TLR5 and not TLR4 [35]. Moreover, CD103^+^ DCs, by producing RA and TGF-β, are responsible for imprinting gut-homing molecules on B cells, and support IgA synthesis [15], [27], [36], [37]. Administration of CTB in the airways results in increased IgA responses, and increased RA-producing capacity as measured by higher ALDH enzyme activity of DC, compared to control conditions. Although in the MedLN a larger proportion of CTB-loaded DCs were CD11b^−^CD103^+^ than CD11b^+^ (figure 5b), expression of ALDH was higher in CD11b^+^ DCs compared to in CD103^+^ DCs after CTB administration (figure 5c). This in contrast to the gut, where the CD103^+^ DCs are the ones that express higher levels of RALDH, and not the CD11b^+^ DCs. Interestingly, and in line with our study, Guilliams et al recently described that in the skin RA production and tolerogenic functions were restricted to CD103^−^ DCs [38]. Furthermore, in the lung, it was suggested recently that CD11b+ DC are more potent in their capacity to induce IgA responses than the CD103+ subset [33]. Thus, CD103 or CD11b may not be specific markers for the identification of specialized DCs subsets with IgA promoting capacity. Although, in our model the increased ALDH activity on the CD11b^+^ DC subset, and the increased IgA after transfer of (CD11b^+^) BM-DC, could suggest that this subset is more important in driving CTB induced IgA responses in the airways, we cannot rule out involvement of CD103^+^ DCs nor other DC subsets. The role of different lung DC subsets and their mechanism of driving humoral IgA responses needs to be further investigated.

CTB can enhance phosphorylation of multiple signaling molecules downstream of TLRs [39], but requires a MyD88-dependent co-activation signal for its IgA inducing capacity. MyD88 is an adaptor molecule downstream of many TLRs [40]. In the gut, DCs are constantly exposed to TLR ligands derived from commensal bacteria, and these signals play a major role in driving IgA responses [2], [10], [14]. Indeed *in vitro* priming of DCs by different MyD88-dependent TLR ligands enhanced IgA production by B cells, but not by MyD88-independent PRR-ligands, such as Poly I:C, Zymosan and Curdlan (figure 2b). Although, CpG DC induce some IgA response by B cells, CTB was not able to enhance this effect. This might be explained by the endosomal localization of TLR9, although this is also the case for TLR 7 and 8 (ligated by CL97) for which we did observe CTB-enhanced IgA induction. Therefore an alternative explanation might be the negative charge of CpG that may influence the IgA-inducing instructions of CTB to the DC.

Importantly, CTB does not only enhance IgA induction by high dose TLR-ligand primed DCs, but also initiates IgA production in case of low dose exposure to MyD88-activating ligands. This is particularly interesting, considering the thought that decreased or altered microbial exposure associated with affluent life style allows uncontrolled inflammatory responses against either innocuous or self-antigens [41]. Indeed, Hilty et al compared the airway microbiota of patients with asthma and controls, and found disturbed microbiota in asthmatic airways [42]. Therefore CTB may have potential in the clinic, by translating insufficient microbial signals into enhanced, and putative protective, IgA levels. In a mouse model, CTB was able to enhance protective mucosal sIgA responses against aero-allergens [8]. Furthermore, in some models CTB can also induce FoxP3^+^ regulatory T cell responses *in vivo* when coupled to specific antigens [43]. Interestingly, oral CTB has already been applied for treatment of Crohn’s disease, reporting decreased disease activity scores [44], which shows the potential use of CTB for hyperinflammatory diseases.

In conclusion, this study demonstrates that the prototypical “mucosal” adjuvant CTB promotes IgA responses by directly instructing DCs to prime IgA responses in B cells, through instructive signals normally found only in the mucosal environment. The CTB instructs mucosal DC priming best when TLR ligands that employ the MyD88 signaling pathway are co-administered. Like in the mucosal environment, CTB-induced IgA induction by non-mucosal DCs, depends on RA and TGF-β. Future studies on CTB are needed to investigate whether CTB equally influences DC driven IgA responses in humans before this work can be employed for boosting IgA responses to allergens in humans.

## Materials and Methods

### Ethics Statement

Mice were housed under SPF-conditions at the animal facilities of the Leiden University Medical Center in Leiden, the Netherlands. All animal studies were performed according to the institutional guidelines and the experimental protocols described in DEC-09028 were approved by the Ethics Committee for Animal Experimentation of the University of Leiden, the Netherlands.

### Animals

Female Balb/c and C57BL/6 mice (6–8 wks) were purchased at Harlan, the Netherlands. MyD88-deficient mice were bred in animal facility (UZ Ghent, Belgium). iNOS-deficient mice were kindly provided by Dr. A Cauwels (VIB Ghent, Belgium).

### Cell Preparation and in vitro Co-culture

BM-DCs were generated from bone marrow cells, as described before [8], and cultured in RPMI 1640 medium containing glutamax (GIBCO), 50 µM 2-mercaptopethanol Signa-Aldrich), 50 µg/ml gentamicin Invitrogen) or sodium-penicillin (Astellas Pharma B.V), streptomycin (0,1 ug/ml, Sigma-Aldrich), and 5% fetal bovine serum (TCM), supplemented with 20 ng/ml recombinant murine granulocyte macrophage-colony stimulating factor (rmGM-CSF, (a gift from K. Thielemans, Vrije Universiteit Brussel, Brussels, Belgium). After 8 days, DCs were pulsed overnight with PBS or LPS (1 or 100 ng/ml) with or without CTB (10 µg/ml, Sigma-Aldrich, St. Louis, MO). In specific experiments, BM-DCs were pulsed with different TLR ligands (Poly I:C 25 µg/ml, LPS 100 ng/ml, Flagellin 1 µg/ml, FLS1 10 µg/ml, Cl97 1 µg/ml, CpG 2.5 µg/ml) or C-type lectin receptor ligands (CTL) (Zymozan 10 µg/ml, Curdlan 150 µg/ml) with and without CTB. To isolate PP DCs, lymph nodes were collected 10–12 days after i.p. injection with 6 million B16 Flt3-L secreting melanoma cells [45] and digested by collagenase (type 3 filtered, Worthington) and DNAse I (Sigma-Aldrich). DCs were isolated using CD11c microbeads (Miltenyi), with a purity of >85% as confirmed by flowcytometry. The remaining cells contained <4% CD19^+^ B cells.

After overnight stimulation, these DCs (1*10^5^) were washed and co-cultured with B cells (1∶1 ratio) and anti-mouse IgM F(ab’)_2_ (10 µg/ml; Jackson Immunoresearch, West Grove, PA) (adapted from [18]) for 7 days. Murine B cells were isolated from spleens using anti-CD19 microbeads (Miltenyi Biotec, Bergisch Gladbach, Germany) with a purity of around 95% as confirmed by flowcytometry. For some experiments, chemical inhibitors were added: RARβ receptor antagonist LE135 (1 µM in DMSO, Tocris Bioscience, Ellisville, MO), or TGFß signaling inhibitor (5 µM, SB-431542, Sigma-Aldrich). Supernatant was collected and immunoglobulin levels determined by ELISA.

### In vivo Mouse Models

OVA (800 µg/mouse) +/− CTB (0.001 or 0.1 µg/mouse, Sigma-Aldrich, St. Louis, MO) intratracheal administration was followed 10 days later by OVA aerosol challenge for 3 consecutive days. One day after the last challenge, lungs were digested with collagenase/DNAse, B cells isolated (CD19+ MACS isolation), and restimulated with LPS (10 µg/ml) for 5 days. Supernatant was collected and IgA production analysed by ELISA. Or AF488-labeled recombinant CTB (10 µg per mouse, Molecular Probes, Eugene, OR) or CTB (0.1 µg/mouse, Sigma-Aldrich, St. Louis, MO) +/− OVA (800 µg/mouse) was administered into the airways. The lung draining lymph nodes and lungs were removed after 36 hrs and digested using collagenase/DNAse [8]; AF488-fluorescence of several cell types or aldehyde dehydrogenase (ALDH) activity (using ALDEFLUOR assay kit, Stemcell Technologies, as described [38]) of DC subsets was studied by flowcytometric staining.

Unpulsed, OVA- (100 µg/ml, containing LPS, Worthington Biochemical Corp., Lakewood, NJ) or OVA+CTB (10 µg/ml) -pulsed BM-DCs (1*10^6^ cells) were administered into the airways, as described [46], while (1 day before until 3 days after) TGFβ and retinoic acid activity was neutralized by i.p injection of LE135 and aTGFβ antibody (or vehicle/isotype control Ab). This was followed 10 days later by OVA aerosol challenge on three consecutive days. One day after the last challenge lungs were isolated, digested using collagenase/DNAse and analysed for presence of IgA positive B cells by flowcytometry.

### RNA Extraction and RT-PCR

BM-DCs were pulsed for 24 hrs and cell pellets were snap-frozen. Subsequently, total RNA was isolated, followed by cDNA synthesis, as described [8]. Quantitative real-time RT-PCR was performed using Brilliant SYBR Green (Stratagene, Santa Clara, CA) in an ABI 7500 RT-PCR machine (Applied Biosystems). Expression was normalized to the housekeeping gene GAPDH, and presented relative to conventional unpulsed BM-DCs. Sequences of primers were used as described [14]. In addition, RALDH2 expression was studied using the following primer sequences: 5′-AGC CCA TTG GAG TGT GT-3′ and 5′-CCA GCC TCC TTG ATG AG-3′.

### Immunoglobulin Measurements

Ig levels (IgA/IgG1/IgG2a/IgE/IgM) were measured by ELISA (BD Biosciences). Detection limit was 2 ng/ml for all Igs.

### Statistical Analysis

To study whether there is a general difference between 3 or more groups, first ANOVA test was performed, followed by Bonferoni post hoc test to compare specific groups or the Mann-Whitney U t-test was used to compare two individual groups. P values less than 0.05 were considered significant. P-values less than 0.05, 0.01 or 0.001 are indicated by one, two or three asterisks, respectively.

## Supporting Information

Figure S1 **BM-DCs were generated by culture in GM-CSF (A and C) or Flt3-L (B).** At day 8, DCs were pulsed with medium, LPS (1 ng/ml), LPS+CTB (10 µg/ml) or CTB only. Thereafter, cells were washed and co-cultured with (A/C) naïve CD43^−^ B cells (ratio 1∶1), or (B/C) with CD19^+^ B cells and anti-IgG/IgM (10 µg/ml). Total CD19^+^ B cells were retrieved by positive selection with CD19 microbeads from splenocytes, while naïve B cells were collected after negative isolation with the CD43^−^ naïve B cell isolation kit (both from Miltenyi). After 7 days, supernatant was collected and IgA levels were measured by ELISA. (C) 48 hrs after the start of the co-culture, B cells were collected and cell pellets snapfrozen. After RNA extraction, RT-QPCR was performed for AID (Primer sequence Forward: 5′-TCC TGC TCA CTG GAC TTC GG-3′, Reverse: 5′-GTG AAC CAG GTG ACG CGG TA-3′) and GAPDH gene expression in CD43^−^ en total CD19^+^ B cells, that were co-cultured with GM-CSF-generated BM-DCs. Mean+sem of 3 or 4 individual experiments are shown * P<0.05, ** P<0.01, *** P<0.001, # P = 0.100.
(PDF)

Figure S2 **Increased LAP expression on LPS+CTB treated BM-DCs. BM-derived DCs were cultured for 8 days with GMCSF, pulsed overnight with PBS, LPS (1 ng/ml) +/− CTB (10 µg/ml) or CTB alone.** LAP expression of PFA fixed and Brefeldin A treated pulsed DCs, by FACS. Geomean is displayed, relative to the expression of unpulsed BM-DC. Mean+sem of 4 individual experiments are shown * P<0.05, ** P<0.01, *** P<0.001.
(PDF)

Figure S3 **Role of BAFF and APRIL in IgA induction by CTB-primed DCs.** BM-derived DCs were cultured and pulsed as described in the legend of figure 1, and then co-cultured with splenic CD19+ B cells (ratio 1∶1) and anti-IgM Fab-fragments (10 µg/ml). After 7 days, IgA levels were determined by ELISA. (A) During co-culture either blocking antibodies against BAFF (0.2 µg/ml, R&D systems), BAFFR3 (2 µg/ml, R&D systems), or isotype controls were added. (B) BM-DCs from B6129S2F1 mice were generated, pulsed and co-cultured with splenic TACI/BCMA^−/−^ B cells (on a B6129S2F1 background) as described. Data from one representative experiment out of 4 are shown. * P<0.05, ** P<0.01, *** P<0.001.
(PDF)

Figure S4 **OVA Worthington contains sufficient LPS to induce IgA in synergy with CTB.** BM-derived DCs were cultured, and pulsed overnight with PBS, OVA (100 µg/ml, containing LPS), LPS free OVA (100 µg/ml, Seikagaku [de Heer, J ex Med 2004]) or LPS (1 ng/ml), either or not in combination with CTB (10 µg/ml) or CTB alone, thereafter cultured with B cells for 7 days as described. Supernatant was collected and IgA production measured by ELISA. Data from one representative experiment out of 2 are shown. * P<0.05, ** P<0.01, *** P<0.001.
(PDF)

Figure S5 **Representative ALDEFLUOR vs CD11c plots of** **figure 5d** **are shown for (A) CD11b+ or (B) CD103^+^ migratory DCs from lung draining LNs.**
(PDF)
