# Supplementary figures and images for: The Mucosal Adjuvant Cholera Toxin B Instructs Non-Mucosal Dendritic Cells to Promote IgA Production Via Retinoic Acid and TGF-β
Source: PLoS One. 2013 Mar 20;8(3):e59822. doi: 10.1371/journal.pone.0059822 (PMC3603891; doi:10.1371/journal.pone.0059822)

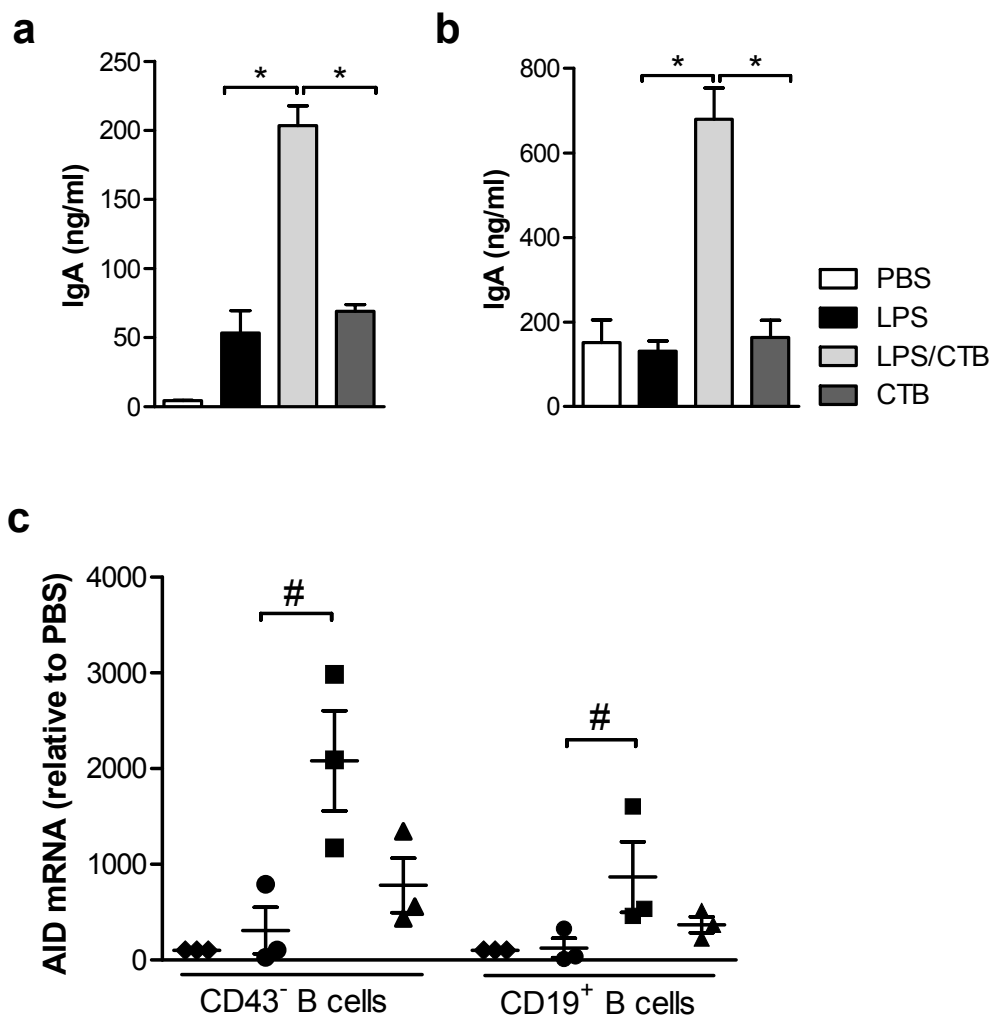

Supplement: Figure S1 — BM-DCs were generated by culture in GM-CSF (A and C) or Flt3-L (B). At day 8, DCs were pulsed with medium, LPS (1 ng/ml), LPS+CTB (10 µg/ml) or CTB only. Thereafter, cells were washed and co-cultured with (A/C) naïve CD43− B cells (ratio 1∶1), or (B/C) with CD19+ B cells and anti-IgG/IgM (10 µg/ml). Total CD19+ B cells were retrieved by positive selection with CD19 microbeads from splenocytes, while naïve B cells were collected after negative isolation with the CD43− naïve B cell isolation kit (both from Miltenyi). After 7 days, supernatant was collected and IgA levels were measured by ELISA. (C) 48 hrs after the start of the co-culture, B cells were collected and cell pellets snapfrozen. After RNA extraction, RT-QPCR was performed for AID (Primer sequence Forward: 5′-TCC TGC TCA CTG GAC TTC GG-3′, Reverse: 5′-GTG AAC CAG GTG ACG CGG TA-3′) and GAPDH gene expression in CD43− en total CD19+ B cells, that were co-cultured with GM-CSF-generated BM-DCs. Mean+sem of 3 or 4 individual experiments are shown * P<0.05, ** P<0.01, *** P<0.001, # P = 0.100. (PDF) [file pone.0059822.s001.pdf]

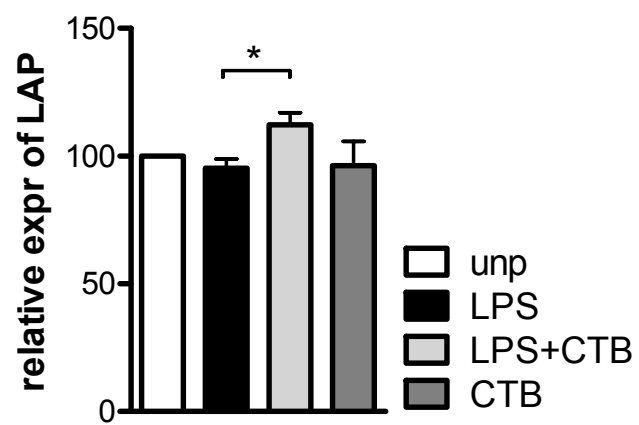

Supplement: Figure S2 — Increased LAP expression on LPS+CTB treated BM-DCs. BM-derived DCs were cultured for 8 days with GMCSF, pulsed overnight with PBS, LPS (1 ng/ml) +/− CTB (10 µg/ml) or CTB alone. LAP expression of PFA fixed and Brefeldin A treated pulsed DCs, by FACS. Geomean is displayed, relative to the expression of unpulsed BM-DC. Mean+sem of 4 individual experiments are shown * P<0.05, ** P<0.01, *** P<0.001. (PDF) [file pone.0059822.s002.pdf]

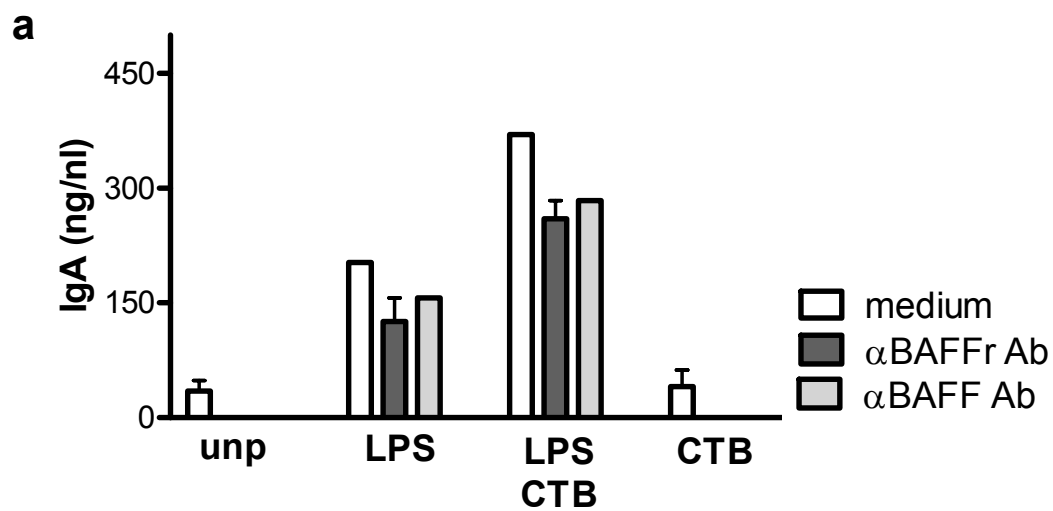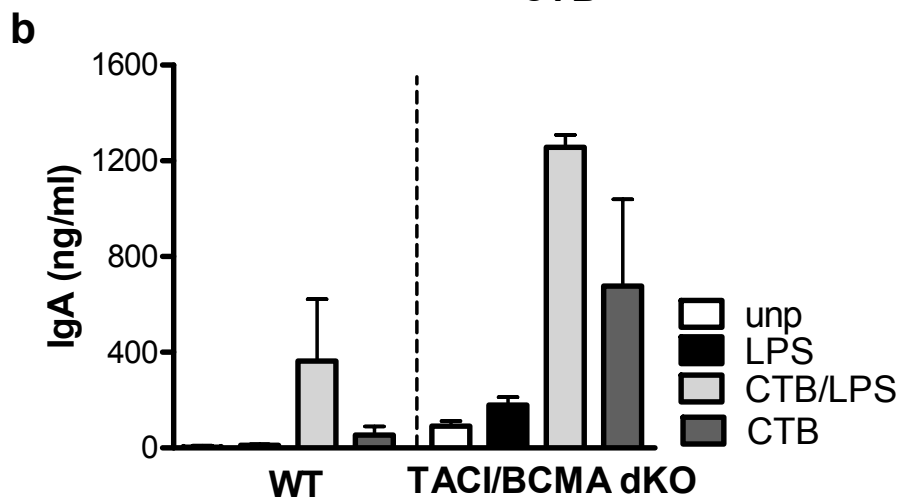

Supplement: Figure S3 — Role of BAFF and APRIL in IgA induction by CTB-primed DCs. BM-derived DCs were cultured and pulsed as described in the legend of figure 1, and then co-cultured with splenic CD19+ B cells (ratio 1∶1) and anti-IgM Fab-fragments (10 µg/ml). After 7 days, IgA levels were determined by ELISA. (A) During co-culture either blocking antibodies against BAFF (0.2 µg/ml, R&D systems), BAFFR3 (2 µg/ml, R&D systems), or isotype controls were added. (B) BM-DCs from B6129S2F1 mice were generated, pulsed and co-cultured with splenic TACI/BCMA−/− B cells (on a B6129S2F1 background) as described. Data from one representative experiment out of 4 are shown. * P<0.05, ** P<0.01, *** P<0.001. (PDF) [file pone.0059822.s003.pdf]

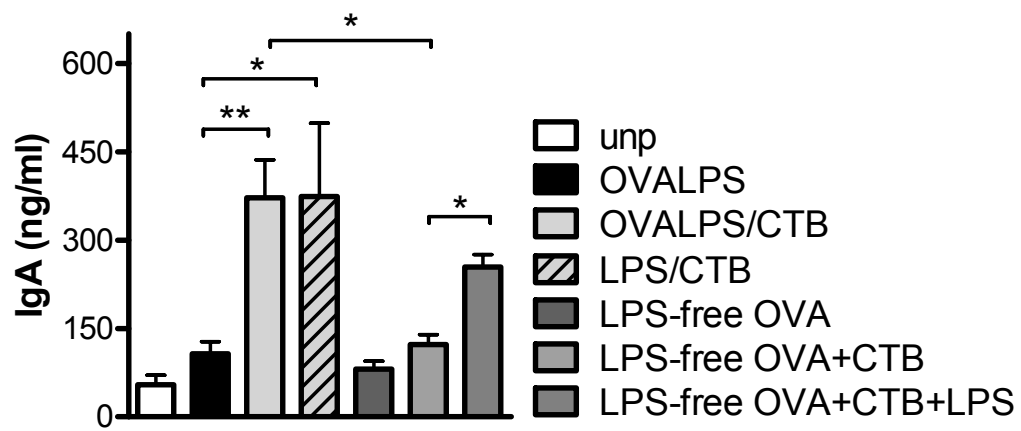

Supplement: Figure S4 — OVA Worthington contains sufficient LPS to induce IgA in synergy with CTB. BM-derived DCs were cultured, and pulsed overnight with PBS, OVA (100 µg/ml, containing LPS), LPS free OVA (100 µg/ml, Seikagaku [de Heer, J ex Med 2004]) or LPS (1 ng/ml), either or not in combination with CTB (10 µg/ml) or CTB alone, thereafter cultured with B cells for 7 days as described. Supernatant was collected and IgA production measured by ELISA. Data from one representative experiment out of 2 are shown. * P<0.05, ** P<0.01, *** P<0.001. (PDF) [file pone.0059822.s004.pdf]

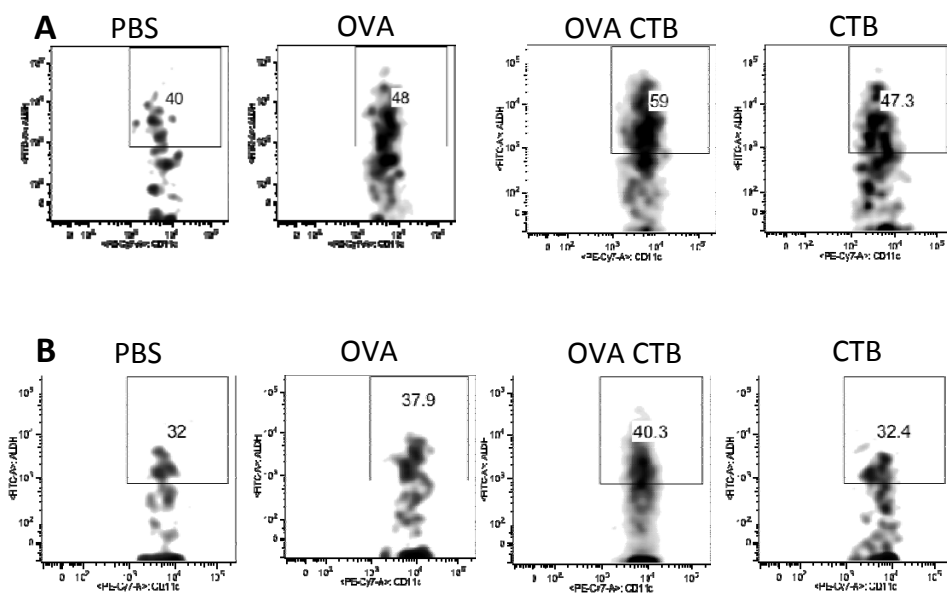

Supplement: Figure S5 — Representative ALDEFLUOR vs CD11c plots of figure 5d are shown for (A) CD11b+ or (B) CD103+ migratory DCs from lung draining LNs. (PDF) [file pone.0059822.s005.pdf]
